# Effects of Government Healthcare Expenditure on Economic Growth Based on Spatial Durbin Model: Evidence from China

**Published:** 2020-02

**Authors:** Xuewei ZHANG, Zong GANG, Xiao DONG

**Affiliations:** 1. School of Economics and Management, Beijing University of Technology, Beijing, China; 2. School of Finance, Henan University of Economics and Law, Zhengzhou, Henan, China

**Keywords:** Healthcare expenditure, Spatial Durbin model, Economic growth

## Abstract

**Background:**

The proportion of government healthcare expenditure in China increases due to rapid economic development in recent years. The growth of government healthcare expenditure can promote physical health improvement of human capitals and thereby facilitate economic growth. Hence, exploring the effects of government healthcare expenditure on economic growth is important.

**Methods:**

Spatial correlation of economic growth under different spatial weights was tested, and the effects of government healthcare expenditure on economic growth were analyzed by constructing a spatial Durbin model with the panel data of 31 provinces in China gathered from 2005 to 2017.

**Results:**

Government healthcare expenditure in China significantly and positively affects economic growth under three spatial weight matrices. The spatial weight of economic distance influences economic growth more significantly compared with the 0–1 spatial weight and the spatial weight of geographical distance. The total and the direct effects of government healthcare expenditure are significantly positive. Furthermore, the direct effects are significant, whereas the indirect effects show different degrees of significance.

**Conclusion:**

The total effect of government healthcare expenditure on economic growth is significant and positive, with direct effects exceeding the indirect ones. Hence, the China’s government must continue to increase financial investment to public health services to promote high-quality economic growth in the country.

## Introduction

**Fig. 1:**
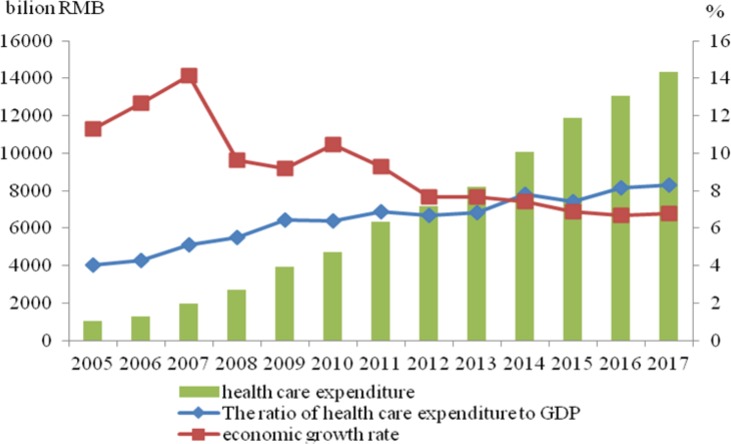
Variation trends of the government healthcare expenditure and economic growth of China from 2005 to 2017

With the gradual expansion of coverage of medical insurance system and the continuous improvement of healthcare services in China, the government healthcare expenditure increased from 2005 to 2017. According to the data published in *China Statistical Yearbook* (1) and *China Health Statistical Yearbook* (2), in 2005, the government healthcare expenditure was 101.555 billion RMB, which accounted for 4.03% of the local government’s general public budget. In the same year, the growth rate of gross domestic product (GDP) was 11.31%. Moreover in 2017, the government healthcare expenditure was 1434.303 billion RMB, which accounted for 8.27% of the local government’s general public budget and was higher than the growth rate of GDP in the same period (6.81%). Figure 1 demonstrates that the proportion of government healthcare expenditure was increasing year by year, and its growth rate was significantly higher than that of GDP. Hence, the absolute value of government healthcare expenditure increased from 2005 to 2017. The government of China (GOC) is exerting great efforts in the medical and healthcare industry.

A reciprocal relationship between healthcare expenditure and economic growth in the short run exists, but this relationship turns into causal in the long run (3). Healthcare expenditure can significantly facilitate economic growth (4–6). Nevertheless, a bidirectional relationship also exists between economic growth and government healthcare expenditure. Related studies tested the positive correlation and stable bidirectional relationship between economic growth and healthcare expenditures by using constraint test method, autoregression lag method, and Kalman filtering model, which find a bidirectional causal relationship between economic growth and healthcare expenditures in the long and the short run (7–9). Moreover, the regional difference in total healthcare costs mostly influences the growth rate of GDP (10).

Several studies evaluated the restriction policy of public healthcare expenditures caused by economic crisis and the acceptance degree of the economic policies from the perspective of citizens and constructed a mean comparison and structural equation model (11–15). These studies concluded that people—though faced with economic crisis—generally oppose the cutting down of healthcare expenditures (11, 12). Previous studies mainly focused on the single or bidirectional relations between healthcare-induced economic growth and government healthcare expenditures and discussed the policies related with government healthcare expenditures (13–15).

Based on the above literature review, many scholars carried out abundant empirical analyses on the economic effect of government healthcare expenditure (16, 17). However, studies concerning the effects of government healthcare expenditure on economic growth through the spatial perspective are rare. With respect to methodology, most associated studies constructed models based on panel data and hardly consider the spatial dependence and spillover effect among different regions. As a result, previous findings lacked enough persuasion and failed to explain the effects of government healthcare expenditure on economic growth.

On this basis, an empirical study on the relationship between the government healthcare expenditure and economic growth of China was carried out by using Spatial Durbin Model (SDM).

## Methodology

### Econometric model

In the present study, a basic model without considering spatial effect was constructed by using traditional panel data. The constructed panel data model is expressed as follows:

(1) $$\mathrm{GD}P_{\mathrm{it}}=\alpha_{0}+\alpha_{1}\mathrm{Hea}l_{\mathrm{it}}+\beta X_{\mathrm{it}}+\mu_{i}+{ɛ}_{\mathrm{it}}$$

where *i* is regions; *t* is year; the explained variable *GDP_it_* refers to *GDP_it_*; *X_it_* is the set of explanatory variable vectors composed of the control variables; *u_i_* is the regional fixed effect; and *ɛ_it_* is the random error term.

Common spatial econometric models include the spatial lag model (SLM), spatial error model (SEM), and SDM. SDM integrates SLM and SEM so it can obtain unbiased estimation, and it is superior to other models. For comprehensive considerations, SDM is constructed to discuss the effects of government healthcare expenditure on economic growth. SDM is expressed as:

(2) Y=ρWY+βX+λWX+ɛ

where *Y* is the economic growth index represented by *GDP* ; *ρ* and *λ* are spatial autocorrelation coefficient; *W* is a spatial weight matrix; *X* is the set of explanatory variable vectors composed of government healthcare expenditure and other control variables that will be introduced in the model; *β* is a set of coefficient vector of explanatory variable; *WX* and *WY* are spatial lag terms of explanatory and explained variables; and *ε* is the random error term. If *ρ* and *λ* ≠ 0, then spatial spillover effect is present.

The effects of government healthcare expenditure on economic growth are explored by constructing SDM. The specific econometric model is expressed as follows:

(3) $$\begin{array}{ll} \mathrm{GD}P_{\mathrm{it}} & =\rho \mathrm{WGD}P_{\mathrm{it}}+\beta_{1}\mathrm{Hea}l_{\mathrm{it}}+\lambda_{1}W_{\mathrm{ij}}\mathrm{Hea}l_{\mathrm{jt}}+\beta_{2}\mathrm{Ca}p_{\mathrm{it}}+\lambda_{2}W_{\mathrm{ij}}\mathrm{Ca}p_{\mathrm{it}}\\ & +\beta_{3}\mathrm{Co}n_{\mathrm{it}}+\lambda_{3}W_{\mathrm{ij}}\mathrm{Co}n_{\mathrm{it}}+\beta_{4}\mathrm{Ex}p_{\mathrm{it}}+\lambda_{4}\mathrm{WEx}p_{\mathrm{it}}+\beta_{5}\mathrm{Hu}m_{\mathrm{it}}\\ & +\lambda_{5}\mathrm{WHu}m_{\mathrm{it}}+\beta_{6}\mathrm{Ur}b_{\mathrm{it}}+\lambda_{6}\mathrm{WUr}b_{\mathrm{it}}+\delta_{i}+u_{t} \end{array}$$

where *i* and *t* are regions and year, respectively; *GDP_it_* refers to the economic growth level; *WGDP_it_* is the spatial lag term of economic growth; *W_it_* is the spatial weight matrix; *Heal_it_* refers to the government healthcare expenditure; *WHeal_it_* is the spatial lag term of government healthcare expenditure; *Cap_it_* is the material capitals, and *WCap_it_* is the spatial lag term of material capitals; *Con_it_* refers to total consumption, and *WCon_it_* refers the spatial lag term of total consumption; *Exp_it_* is the total exports, and *WExp_it_* is the spatial lag term of total exports; *Hum_it_* is the human capitals, and *WHum_it_* means the spatial lag term of human capitals; *Urb_it_*refers to the urbanization level, and *WUrb_it_* is the spatial lag term of urbanization level; *δ_i_* and *μ_t_* are the individual and the time effects, respectively.

### Estimation method

The regression coefficients cannot measure spatial spillover effect of explanatory variable directly. Hence, LeSage and Pace proposed the partial derivatives method of spatial regression model (18). Specific deduction process is shown as follows:

(4) $$(I_{n}-\rho W)Y=\beta X+\lambda \mathrm{WX}+ɛ$$

(5) $$Y={\left(I_{n}-\rho W\right)}^{-1}(I_{1}\beta +W\lambda )X+{\left(I_{n}-\rho W\right)}^{-1}ɛ$$

(6) $$Y=\sum_{s=1}^{t}K(W)X_{s}+M(W)ɛ$$

(7) $$M(W)={\left(I_{n}-\rho W\right)}^{-1}=\mathrm{In}+\rho W+\rho^{2}W^{2}+\rho^{3}W^{3}+\ldots$$

(8) $$K_{s}(W)=M(W)(I_{n}\beta_{s}+W\lambda_{s})$$

where *t* is number of explanatory variables; *I_n_* is the *n*-order unit matrix; *β_s_* is the regression coefficient of the *s^th^* explanatory variable; *λ_s_* is the regression coefficient of the *s^th^* variable in *WX*.

To elaborate effects of *K_s_*(*W*), Eq. [6] was transformed into Eq.[9]. Therefore, *Y_i_* in the region *i* (*i*= 1, 2, 3, …, *n*) also can be expressed as the Eq. [10].

(9) $$\left[\begin{array}{c} Y_{1}\\ Y_{2}\\ \ldots \\ Y_{n} \end{array}\right]=\sum_{s=1}^{t}\left[\begin{array}{cccc} K{\left(W\right)}_{11} & K{\left(W\right)}_{12} & \ldots & K{\left(W\right)}_{1n}\\ K{\left(W\right)}_{21} & K{\left(W\right)}_{22} & \ldots & K{\left(W\right)}_{2n}\\ \ldots & \ldots & \ldots & \ldots \\ K{\left(W\right)}_{n1} & K{\left(W\right)}_{n2} & \ldots & K{\left(W\right)}_{\mathrm{nn}} \end{array}\right]\left[\begin{array}{c} X_{1s}\\ X_{2s}\\ \ldots \\ X_{\mathrm{ns}} \end{array}\right]+M(W)ɛ$$

(10) $$Y_{i}=\sum_{s=1}^{t}\left[K_{s}(W)X_{1s}+K_{s}(W)X_{2s}+\ldots +K_{s}(W)X_{\mathrm{ns}}\right]+M{\left(W\right)}_{i}ɛ$$

According to the Eq. [10], partial deviation of *Y_i_* in relative to explanatory variable *X_js_* (the *s^th^* explanatory variable) of other regions (*j*) was calculated, thus obtaining Eq. [11]. Similarly, Eq. [12] can be obtained by calculating the partial deviation of *Y_i_* in relative to explanatory variable *X_is_* in the local area:

(11) $$\frac{\partial Y_{i}}{\partial X_{\mathrm{js}}}=K_{s}{\left(W\right)}_{\mathrm{ij}}$$

(12) $$\frac{\partial Y_{i}}{\partial X_{\mathrm{is}}}=K_{s}{\left(W\right)}_{\mathrm{ii}}$$

where *K_s_*(*W*)*_ij_* measures effects of *X_js_* on *Y_i_*, and *K_s_*(*W*)*_ii_* measures effects of *X_is_* on *Y_i_*. According to the Eqs. [11] and [12], the explanatory variables of a region may influence explained variables in other regions and the local region. The effects on explained variables in other regions are called indirect effect, whereas the effects on explained variables in local regions are called direct effect. The sum of indirect effects and direct effects is called the total effect.

### Descriptive statistics of variables

1) ***Explained variable***

Economic growth is measured by *GDP* (100 million RMB). Economic growth means that a high-level GDP can provide high-quality commodities and services as well as improved residence environment by high-level GDP.

2) ***Explanatory variable***

Government healthcare expenditure (*Heal*) is measured by the government healthcare expenditures of provinces, cities, and municipalities. According to the endogenous growth theory (19), government public expenditure is an important factor that determines economic growth.

3) ***Control variables***

Material capitals (*Cap*) is measured by the fixed investment (100 billion RMB) of provinces, cities, and municipalities. Total consumption (*Con*) is measured by the total retail sales (100 billion RMB) of consumer goods of provinces, cities, and municipalities in the current year. Total export (*Exp*) is measured by the total good export volume (100 billion RMB) of provinces, cities, and municipalities in the current year. Human capital (*Hum*) is measured by the number of college students in 10,000 people of provinces, cities, and municipalities. Human capital is viewed as an indispensable element that determines economic growth (20, 21). In urbanization level (*Urb*), the proportion of urban population in total population (%) was used as a measurement index of urbanization level. Table 1 summarizes definition of variables and descriptive statistics.

**Table 1:** Definition of variables and descriptive statistics

***Name***	***Signs***	***Calculation methods***	***Means***	***Minimum***	***Maximum***
Explained variable					
Economic growth	*GDP*	Logarithm of GDP	9.241	5.516	11.404
Explanatory variable					
Healthcare expenditures	*Heal*	Logarithm of healthcare expenditures	4.901	1.686	7.175
Control variables					
Material capitals	*Cap*	Logarithm of fixed asset investment	8.815	5.279	10.918
Total consumption	*Con*	Logarithm of total retail sales of consumer goods	8.202	4.291	10.551
Total export	*Exp*	Logarithm of total exports	6.901	2.606	10.646
Human capital	*Hum*	Logarithm of number of college students	13.175	9.851	14.516
Urbanization rate	*Urb*	Proportion of urban population	0.520	0.226	0.896

### Data

In the present study, data are collected from previous *China Statistical Yearbook*, *China Health Statistical Yearbook*, *China Population Statistical Yearbook*, *China Population and Employment Statistical Yearbook*, and *Finance Year Book of China*. The balanced panel data of 31 provinces from 2005 to 2017 are gained after data review. To ensure the robustness and reliability of empirical results, the spatial weight matrix (*W*) must be set before parameter estimation.

1. The setting of 0–1 spatial weight matrix observes to the first law of economic geography. If a common border exists between regions *i* and *j*, then the spatial weight matrix is 1. Otherwise, it is 0. Such 0–1 spatial weight matrix was recorded as *W*_1_.
2. Spatial weight matrix of geographical distance is set by the reciprocal of square of longitude and latitude distance of prefecture-level cities in different provinces, which is recorded as *W*_2_. $W_{\mathrm{ij}}=1/D_{\mathrm{ij}}{}^{2}$, where *D_ij_* refers to the distance between two prefecture-level cities.
3. Spatial weight matrix of economic distance considers the economic and geographical correlations among different regions. The expression is $W=W_{2}{}^{*}\;\mathrm{diag}({\bar{y}}_{1}/\bar{y},{\bar{y}}_{2}/\bar{y},{\bar{y}}_{3}/\bar{y})$, where the economic variable *ȳ_i_* represents the mean per capita GDP of the region *I*, and *ȳ* is the mean per capita GDP of all regions.

## Results

### Spatial correlation test

Exploratory spatial data analysis is an important research field in spatial econometrics. In this study, the spatial distribution pattern of economic growth is described by using ESDA method, and spatial autocorrelation is reflected by the global Moran's I index (22). The expression of global Moran's I index is as follows:

(13) Moran's I=∑i=1n∑j=1nWij(Yi−Y¯)(Yj−Y¯)S2∑i=1n∑j=1nWij

(14) $$S^{2}=\frac{1}{n}\sum_{i=1}^{n}{\left(Y_{i}-Y\right)}^{2},\;\bar{Y}=\frac{1}{n}\sum_{i=1}^{n}Y_{i}$$

where. *Y_i_* and *Y_j_* are economic growth levels of provinces *i* and *j*, respectively; and *W_ij_* is the spatial weight matrix, and its value can present regular variations in accordance to geographical distance. The value of *Moran’s* I ranges from −1 to 1. If *Moran’s* I > 0, then a positive spatial autocorrelation exists. If *Moran’s* I < 0, then negative spatial autocorrelation exists. The spatial correlation strengthens when absolute value increases. If *Moran’s* I = 0, then no spatial correlation exists.

The measurement of the Moran’s I index of provincial economic growth of China from 2005 to 2017 (Table 2) shows that the Moran’s I index is positive and fluctuates slightly. The Moran’s I values are relatively similar under the 0–1 spatial weight matrix and spatial weight matrix of economic distance. However, the Moran’s I value under the spatial weight matrix of geographical distance is lower than those under the rest two spatial weight matrixes, and it declines slowly.

**Table 2:** Moran’s I index

***Year***	***W_1_***			***W_2_***			***W_3_***		
	I	z	p	I	z	p	I	z	p
2005	0.280	3.011	0.001	0.223	2.002	0.023	0.228	1.375	0.085
2006	0.275	2.957	0.002	0.217	1.954	0.025	0.221	1.338	0.090
2007	0.275	2.962	0.002	0.214	1.936	0.026	0.216	1.314	0.094
2008	0.274	2.960	0.002	0.214	1.939	0.026	0.215	1.311	0.095
2009	0.280	3.015	0.001	0.217	1.959	0.025	0.218	1.322	0.093
2010	0.278	3.009	0.001	0.217	1.965	0.025	0.216	1.318	0.094
2011	0.276	2.993	0.001	0.214	1.949	0.026	0.210	1.289	0.099
2012	0.270	2.941	0.002	0.210	1.921	0.027	0.204	1.260	0.104
2013	0.268	2.918	0.002	0.209	1.910	0.028	0.204	1.255	0.105
2014	0.269	2.925	0.002	0.209	1.908	0.028	0.204	1.256	0.104
2015	0.282	3.039	0.001	0.216	1.961	0.025	0.211	1.290	0.099
2016	0.297	3.180	0.001	0.229	2.061	0.020	0.227	1.371	0.085
2017	0.270	2.654	0.004	0.240	2.140	0.016	0.233	1.399	0.081

Notes: *W*_1_ is the 0–1 spatial weight; *W*_2_ is the spatial weight of geographical distance; and *W*_3_ is the spatial weight of economic distance, hereinafter the same.

### Estimation results of full-sample SDM

SDM includes two forms of effects, namely, spatial individual fixed effect and spatial individual random effect. The estimated two forms of economic effects of government healthcare expenditure under three weights were gained through the maximum likelihood estimation. Results are shown in Table 3. According to estimation results of SDM in Table 3, the goodness of fit between individual fixed and individual random effect models under three spatial weight matrixes is relatively similar. However, the log likelihood of the fixed effect model is higher than that of the random effect model. Therefore, the spatial Durbin individual fixed effect model was applied to analyze the economic spatial spillover effect of government healthcare expenditure. Under three spatial weights, the spatial model coefficient (*ρ*) under three spatial weights are significantly positive. Thus, the spatial spillover effects of provincial economic growth in China are very significant.

**Table 3:** Estimation results of full-sample SDM

***Variables***	***W_1_***		***W_2_***		***W_3_***	
	SDM-RE	SDE-FE	SDM-RE	SDE-FE	SDM-RE	SDE-FE
*Heal*	0.048^*^ (0.028)	0.076^***^ (0.027)	0.041 (0.026)	0.068^***^ (0.026)	0.055^**^ (0.026)	0.081^***^ (0.026)
*Cap*	0.101^***^ (0.017)	0.129^***^ (0.019)	0.120^***^ (0.017)	0.146^***^ (0.018)	0.120^***^ (0.017)	0.149^***^ (0.018)
*Con*	0.732^***^ (0.037)	0.548^***^ (0.079)	0.742^***^ (0.039)	0.510^***^ (0.084)	0.726^***^ (0.041)	0.480^***^ (0.085)
*Exp*	0.011 (0.010)	0.0056 (0.010)	0.015 (0.010)	0.006 (0.010)	0.013 (0.010)	0.005 (0.010)
*Hum*	0.063^*^ (0.035)	0.042 (0.040)	0.027 (0.035)	0.024 (0.041)	0.033 (0.036)	0.032 (0.041)
*Urb*	−0.056 (0.140)	−0.33^*^ (0.173)	−0.090 (0.136)	−0.374^**^ (0.169)	−0.191 (0.138)	−0.438^***^ (0.167)
*ρ*	0.373^***^ (0.058)	0.323^***^ (0.060)	0.510^***^ (0.063)	0.507^***^ (0.064)	0.499^***^ (0.064)	0.481^***^ (0.065)
*W×Heal*	−0.013 (0.034)	0.009 (0.037)	0.001 (0.038)	0.018 (0.044)	−0.007 (0.041)	0.014 (0.051)
*W×Cap*	−0.011 (0.033)	0.007 (0.036)	−0.048 (0.038)	−0.039 (0.040)	−0.045 (0.039)	−0.041 (0.042)
*W×Con*	−0.358^***^ (0.063)	−0.201^**^ (0.097)	−0.503^***^ (0.069)	−0.344^***^ (0.108)	−0.499^***^ (0.069)	−0.293^**^ (0.114)
*W×Exp*	0.053^***^ (0.015)	0.067^***^ (0.015)	0.037^*^ (0.020)	0.051^***^ (0.019)	0.058^***^ (0.022)	0.078^***^ (0.021)
*W×Hum*	−0.059 (0.055)	−0.173^**^ (0.076)	0.007 (0.067)	−0.136 (0.095)	−0.027 (0.076)	−0.197^*^ (0.108)
*W×Urb*	−0.419 (0.285)	−0.488 (0.329)	−0.097 (0.286)	0.271 (0.379)	0.051 (0.268)	0.189 (0.379)
*N*	403	403	403	403	403	403
*R^2^*	0.987	0.961	0.989	0.964	0.989	0.963
*Hausman likelihood*	−14.14		−11.12		−3.44	
	539.135	626.765	546.566	633.778	544.463	633.701

Notes: numbers in brackets reflect standard error.

^***^, ^**^, and ^*^ are significance at the 1%, 5%, and 10% levels

The relationship between economic growth and government healthcare expenditure must be investigated from the perspective of spatial economy. The direct effects of government healthcare expenditure, material capital, and total consumption on economic growth are significantly positive under three spatial weight matrixes. The estimation results of government healthcare expenditure under the spatial weight matrix of economic distance are optimal. The estimation results of the three spatial weight matrixes are basically consistent. Therefore, the estimation results of spatial Durbin individual fixed effect model are robust. Therefore, the economic spatial spill-over effects of government healthcare expenditure are further decomposed via the spatial regression with partial derivatives method. Results are shown in Table 4.

**Table 4:** Spillover effect decomposition of SDM

***Model variables***	***Direct effect***			***Indirect effect***			***Total effect***		
	*W_1_*	*W_2_*	*W_3_*	*W_1_*	*W_2_*	*W_3_*	*W_1_*	*W_2_*	*W_3_*
*Heal*	0.080^***^ (0.026)	0.074^***^ (0.025)	0.086^***^ (0.025)	0.045 (0.042)	0.098 (0.069)	0.092 (0.079)	0.126^***^ (0.039)	0.173^**^ (0.068)	0.179^**^ (0.076)
*Cap*	0.132^***^ (0.018)	0.149^***^ (0.018)	0.151^***^ (0.018)	0.067 (0.044)	0.066 (0.073)	0.055 (0.073)	0.200^***^ (0.046)	0.215^***^ (0.077)	0.206^***^ (0.077)
*Con*	0.555^***^ (0.072)	0.510^***^ (0.077)	0.483^***^ (0.077)	−0.037 (0.102)	−0.169 (0.141)	−0.119 (0.145)	0.517^***^ (0.077)	0.341^***^ (0.122)	0.364^***^ (0.122)
*Exp*	0.010 (0.009)	0.011 (0.009)	0.012 (0.009)	0.096^***^ (0.018)	0.106^***^ (0.029)	0.151^***^ (0.032)	0.108^***^ (0.019)	0.118^***^ (0.030)	0.163^***^ (0.032)
*Hum*	0.028 (0.037)	0.011 (0.037)	0.015 (0.037)	−0.228^**^ (0.099)	−0.237 (0.172)	−0.331^*^ (0.189)	−0.199^*^ (0.103)	−0.226 (0.177)	−0.316 (0.194)
*Urb*	−0.379^**^ (0.166)	−0.363^**^ (0.164)	−0.438^***^ (0.163)	−0.833^*^ (0.434)	0.153 (0.682)	−0.033 (0.654)	−1.212^***^ (0.453)	−0.210 (0.711)	−0.472 (0.687)

Notes: numbers in brackets reflect standard error.

^***^, ^**^, and ^*^ are significance at the 1%, 5%, and 10% levels

### Decomposition of economic spatial spillover effects of government healthcare expenditure

SDM involves the spatial lag terms of dependent and independent variables. Hence, the total effects of independent variables on dependent variables are decomposed into direct and indirect effects via the effect decomposition technique. The total economic, direct, and indirect effects of government healthcare expenditure are estimated by using the “partial derivatives method” proposed by Lesage and Pace. Results are shown in Table 4. The government healthcare expenditure passes through the 1% significance test under three spatial weights. The direct effect of government healthcare expenditure is the highest under the spatial weight of economic distance. Therefore, government healthcare expenditure will promote local economic development significantly, and this positive effect reaches the maximum after considering economic factors. Under three spatial weights, the indirect effects of government healthcare expenditure on economic growth are positive but not very significant because government healthcare expenditure belongs to public expenditure.

Furthermore, government healthcare expenditure mainly influences local economic development, with only few effects on economic development in other regions. The total economic effects of government healthcare expenditure are significantly positive effects, and the direct effect coefficient is higher than the indirect effect coefficient. Therefore, government healthcare expenditure is beneficial to not only increasing market management level and public health quality of the entire society but also to perfecting system supply. Therefore, government healthcare expenditure can directly promote local economic growth.

### Regional heterogeneity of economic effects of government healthcare expenditure

China has extremely unbalanced regional economic development levels, and different regions have various healthcare expenditures. Whether regional difference in contributions of healthcare expenditures to economic growth exists or not still remains to be analyzed through the effects of government healthcare expenditure in East China (11 provinces), Central China (8 provinces), and West China (12 provinces) on economic growth. As shown in Table 5, government healthcare expenditure in East China negatively and insignificantly affects economic growth. The effects of government healthcare expenditure on economic growth in Central China pass through the significance test under 1% level. The effects of healthcare expenditure on economic growth in West China are positive but insignificant. The spatial spillover effects of economic growth in East and Central China pass the significance test under 1% level. Thus, these regions have certain spatial spillover effects of economic growth. However, West China fails in the significance test.

**Table 5:** Estimation results of SDM in different regions

	***East China***	***Central China***	***West China***
*Heal*	−0.034 (0.037)	0.191^***^ (0.052)	0.056 (0.044)
*Cap*	0.254^***^ (0.019)	−0.001 (0.038)	0.197^***^ (0.035)
*Con*	0.314^**^ (0.133)	0.944^***^ (0.191)	0.695^***^ (0.106)
*Exp*	−0.046 (0.032)	0.057^***^ (0.016)	−0.001 (0.012)
*Hum*	−0.078 (0.071)	−0.138^*^ (0.074)	0.019 (0.059)
*Urb*	−0.890^***^ (0.235)	−2.451^***^ (0.527)	0.416 (0.277)
*ρ*	0.324^***^ (0.084)	0.104 (0.099)	0.406^***^ (0.091)
*W×Heal*	−0.099 (0.083)	0.060 (0.074)	0.079 (0.066)
*W×Cap*	−0.162^***^ (0.061)	−0.051 (0.061)	−0.123 (0.076)
*W×Con*	0.433^**^ (0.185)	−0.431^**^ (0.195)	−0.502^**^ (0.201)
*W×Exp*	0.184^***^ (0.047)	0.103^***^ (0.025)	0.036^*^ (0.020)
*W×Hum*	0.195 (0.140)	−0.432^**^ (0.179)	0.013 (0.169)
*W×Urb*	−1.614^***^ (0.592)	1.697^***^ (0.625)	−0.534 (0.979)
*N*	143	104	156
*R2 likelihood*	0.992	0.993	0.991
	265.984	190.903	256.010

Notes: numbers in brackets reflect standard error.

^***^, ^**^, and ^*^ are significance at the 1%, 5%, and 10% levels

## Discussion

Empirical analysis concludes that the healthcare expenditure of the Chinese government significantly and positively affects economic growth. The direct effects outweigh the indirect effect. However, the economic effects of government healthcare expenditure have a regional heterogeneity.

First, Table 3 demonstrates that effects of government healthcare expenditure on GDP are significantly positive under three spatial weight matrixes. Moreover, the regression coefficient of government health expenditure is the largest under the spatial weight matrix of economic distance. The regression coefficient of government healthcare expenditure is the highest under the spatial weight matrix of economic distance. This result conforms to another study based on an empirical analysis of systematic generalized matrix model that the government public healthcare expenditures and economic growth are positively correlated (23).

Second, Table 4 shows that the estimation coefficients of the direct, indirect, and total effects of the government healthcare expenditure of China on economic growth are all positive. Government healthcare expenditure acts on the production of high-quality labor force, thus facilitating economic and social development and creating a positive spatial spillover effect. This finding disagrees with the conclusion of Wang based on the spatial econometric model: the indirect effects of most variables of the government healthcare expenditure are insignificant (24).

Third, Table 5 shows that the effects of government healthcare expenditure on economic growth have regional heterogeneity, which is attributed to different supports to healthcare affairs and different investment to subjects demanding for healthcare services in different local governments. These conclusions conform to the findings of Palència et al. based on a trend test, that is, imbalance exists between government healthcare expenditures and economic development. Governments at all levels are held responsible for guaranteeing the supply and supervision of medical and healthcare development (25).

However, this study also has certain limitations. The time span in this study is 13 years, so it is difficult to comprehensively analyze the evolution law of Chinese government health expenditure and economic growth. Future studies about measurement of economic effects of government healthcare expenditure based on spatial econometric model can involve the time dimension and apply a spatial–temporal model to discuss economic effects of government healthcare expenditure

## Conclusion

First, the effects of government healthcare expenditure of China on economic growth are significantly positive under three spatial weight matrixes. Second, the total and the direct effects of government healthcare expenditure are significantly positive. The direct effects of rest control variables are relatively significant. Third, the effects of government healthcare expenditure on economic growth have regional heterogeneity.

## Ethical considerations

Ethical issues (Including plagiarism, Informed Consent, misconduct, data fabrication and/or falsification, double publication and/or submission, redundancy, etc.) have been completely observed by the authors.
